# Characterization of metalloprotease and serine protease activities in batch CHO cell cultures: control of human recombinant IFN-γ proteolysis by addition of iron citrate

**DOI:** 10.1186/1753-6561-5-S8-P115

**Published:** 2011-11-22

**Authors:** Marie-Françoise Clincke, Emmanuel Guedon, Frances T Yen, Virginie Ogier, Jean-Louis Goergen

**Affiliations:** 1 Laboratoire Réactions et Génie des Procédés UPR-CNRS 3349, ENSAIA-INPL, Nancy-Université, 54505 Vandoeuvre-lès-Nancy, France; 2 Lipidomix (EA4422), ENSAIA-INPL, Nancy-Université, 54505 Vandoeuvre-lès-Nancy, France; 3 Genclis SAS, 54505 Vandoeuvre-lès-Nancy, France

## Background

During the production of any recombinant proteins, an evaluation of the product quality is crucial. Proteolytic events may occur during the process and could influence the product quality. Indeed, proteolysis is an unpredictable process and relatively little is know regarding the proteolytic enzymes produced and released by mammalian cells. In fact, proteases originating from the host cell line cannot be avoided in cell culture. Due to regulatory and safety prospects, the addition of serum, fetuin or albumin that usually limit protease activities, is not desirable. Thus, in serum-free cultures of mammalian cells, control of protease activity constitutes a major challenge.

In the present work, the presence of proteases and their effect on quality of IFN-γ produced by a recombinant CHO cell line cultivated in a stirred-tank bioreactor were studied. Whereas the quality of IFN-γ remained constant during the CHO cell cultures performed in BDM medium, IFN-γ proteolysis was observed when cultures were carried out in RPMI medium with serum [1,2].

## Materials and methods

IFN-γ producing CHO cell lines (CHO 320: dhfr^+^, α2,6 ST^-^) were grown in RPMI supplemented with 5% serum and in BDM medium [3]. Whereas RPMI is a classical medium containing serum, BDM is a chemically defined medium without any proteins or serum addition, but supplemented with 0.1% pluronic F-68, 750 µM ethanolamine and 500 µM iron citrate.

Batch cultures were performed in stirred-tank bioreactor (Inceltech, SGI). Dissolved oxygen concentration was set at 50% of air saturation. Agitation rate used was 50 rpm; pH and temperature were set at 7.2 and 37°C respectively.

Glycosylation macroheterogeneity of IFN-γ was characterized by Western Blot (Amersham Biosciences).

Gelatinase and caseinase activities were performed using zymography. Cell-free culture supernatants were concentrated 2-fold on a 10-kDa cutoff filter. Then, the concentrated samples were instantly mixed 3:1 with nonreducing electrophoresis sample buffer (4.8 mL H_2_O; 1.2 mL Tris-HCl 0.5 M pH 6.8; 2 mL SDS 10%; 1 mL glycerol; 0.5 mL bromophenol blue) and loaded on the zymogram gels. The SDS-PAGE gels (10% acrylamide) contained either 0.05% caseine or 0.1% gelatin. The gels were run at 25 mA for 1h. To remove SDS, the gels were soaked twice for 30 min in 2% Triton X-100 on a shaker, then washed in distilled H_2_O followed by an 24h incubation in developing buffer (0.5 M Tris, pH 7.4, 1 µM Zn^2+^ and 5 mM Ca^2+^). Inhibitor supplementations were also performed using EDTA^a^, PMSF^b^ and Complete inhibitor Cocktail^c^. Visualization of protease activity was carried out by incubation for around 3h in a Coomassie blue solution.

^a^EDTA (ethylenediaminetetraacetic acid) = inhibitor of metalloprotease activities

^b^Complete, Mini, EDTA-free (Roche) = serine and cysteine proteases inhibitor cocktail

^c^PMSF (phenylmethylsulfonyl fluoride) = inhibitor of serine protease activities

## Results

**Figure 1:**
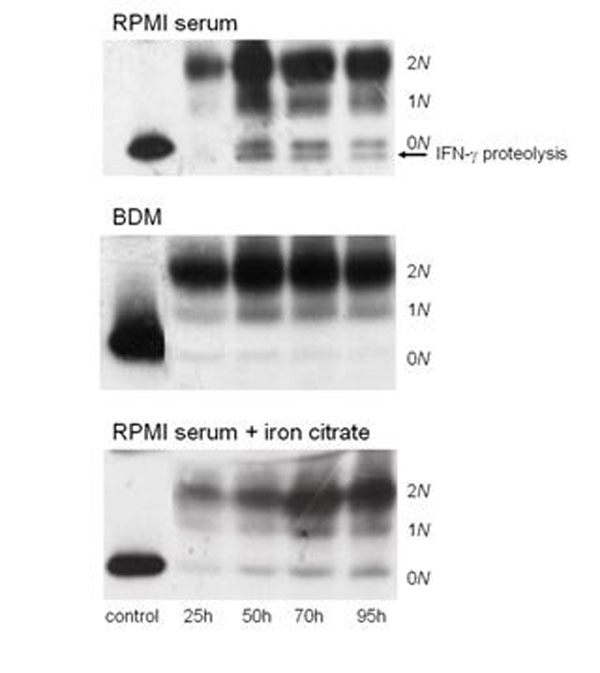
Western blot analysis of excreted IFN-γ produced by CHO cells cultivated in various media; RPMI serum, BDM and RPMI serum supplemented with iron citrate

CHO cell cultures producing human recombinant IFN-γ were cultivated in stirred-tank bioreactor in both RPMI supplemented with 5% serum and BDM media. In both media, three major molecular weight variants (2N, 1N, 0N) were detected during the process with a majority of IFN-γ doubly-glycosylated (2N) whatever the medium used. However, using the RPMI medium with serum, IFN-γ proteolysis was observed during the whole culture (Figure 1).

To determine the protease activities during CHO cell cultures performed in both media, zymogram gels containing either gelatin or casein were performed. Among the 5 caseinase activities detected when CHO cell cultures were performed with serum (Table 1), only the protease activities present all over the process could be potentially involved in the IFN-γ proteolysis (220, 90 and 85 kDa). In addition, 2 gelatinase activities were detected during the whole process (90 and 65 kDa). When CHO cell cultures were carried out using protein-free BDM medium, 2 caseinase activities (90 and 85 kDa) and 1 gelatinase activity (90 kDa) were detected. To determine the type of protease activities present, different inhibitors were used in zymogram gels containing either casein or gelatin. EDTA inhibited all the gelatinase activities, identifying these enzymes as metalloproteases, whereas PMSF and Complete inhibitor Cocktail inhibited all the caseinase activities, classifying these enzymes as serine proteases (data not shown).

**Table 1:** Protease activities detected by zymography during CHO cell cultures performed in RPMI medium with serum and BDM medium

Molecular Weight (kDa)	RPMI medium with serum	BDM medium
Caseinase activities	220, 140, 90, 85 and 40 kDa	90 and 85 kDa
Gelatinase activities	95, 90 and 65 kDa	90 kDa

Compositions of both BDM and RPMI with serum were compared and 3 components which are present in BDM but completely absent in RPMI were identified. These 3 components are pluronic F-68 (PF-68), iron citrate and ethanolamine. Interestingly, addition of iron citrate in the developing buffer of zymogram gels allowed to inhibit the metalloprotease activities, most likely by blocking the Zinc atom in the catalytic domain (data not shown). CHO cell cultures were then performed in RPMI serum supplemented with iron citrate. Addition of iron citrate to RPMI serum allowed to minimized IFN-γ proteolysis (Figure 1). Furthermore, when CHO cell cultures were performed in BDM medium without iron citrate during the first 30 hours of the culture, IFN-γ proteolysis was detected (data not shown). Therefore, IFN-γ proteolysis has at least one cellular origin and the protease responsible for IFN-γ proteolysis is most likely a metalloprotease (Molecular Weight close to 90 kDa).

## Conclusions

Using zymogram analysis, gelatinase and caseinase activities in CHO batch cultures performed with or without serum were detected, and belong most likely to the metalloprotease and serine protease families. When cultures were carried out in RPMI with serum, a degradation of recombinant IFN-γ was observed, while no IFN-γ proteolysis was detected in culture performed with BDM medium. Furthermore, our results showed that despite the medium used (RPMI, BDM, with or without serum), addition of iron minimized IFN-γ proteolysis, probably due to the inhibition of a 90 kDa metalloprotease activity. Thus, we demonstrated that the addition of iron citrate can be advantageously considered for industrial processes to prevent the proteolysis of a recombinant protein, in particular if one or several metalloproteases are present in the culture.
